# The role of psychological factors in the career of the independent dancer

**DOI:** 10.3389/fpsyg.2015.01688

**Published:** 2015-10-30

**Authors:** Imogen Aujla, Rachel Farrer

**Affiliations:** Department of Performing Arts and English, University of Bedfordshire, Bedford, UK

**Keywords:** motivation, mental skills, dancers, dancing, positive psychology

## Abstract

Previous research indicates that psychological factors such as motivation and mental skills play an important role in relation to performance and to negotiating talent development stages. However, little is known about these factors in dance, particularly with regard to the independent dancer whose career may involve multiple roles, varied work patterns, and periods of instability. The aim of this study was to explore dancers’ motivation to work in an independent capacity, and the extent to which dancers’ psychological characteristics and skills enabled them to navigate a career in this demanding sector. In-depth semi-structured interviews were conducted with 14 dancers at different stages of their careers. Interviews were transcribed verbatim and content analyzed. Analysis revealed that the dancers were intrinsically motivated and highly committed to the profession. Working in the independent sector offered dancers opportunities for growth and fulfillment; they appreciated the autonomy, flexibility and freedom that the independent career afforded, as well as working with new people across roles and disciplines. In order to overcome the various challenges associated with the independent role, optimism, self-belief, social support, and career management skills were crucial. The mental skills reported by the participants had developed gradually in response to the demands that they faced. Therefore, mental skills training could be invaluable for dancers to help them successfully negotiate the independent sector.

## Introduction

Dance psychology is a burgeoning area of research within the broader field of dance medicine and science. Researchers have investigated a range of topics in dance psychology, such as performance anxiety (e.g., Walker and Nordin-Bates, 2010) and injury psychology (e.g., Noh et al., 2007). A limited number of studies have also begun to address the role of psychological factors within talent or career development (e.g., Critien and Ollis, 2006; Aujla et al., 2014); however, little is known about factors such as motivation, psychological characteristics and mental skills and the extent to which they are required, developed and employed by independent (freelance) dancers as they negotiate an unpredictable profession.

The term “independent dancer” is commonly used to describe professional contemporary dancers in the UK who frequently transition between jobs and projects, typically without a long-term commitment to a company or organization. As Clarke (1997, p. 2) explained, “…these dance artists work as freelance entrepreneurs, often juggling many roles simultaneously and taking their expertise into numerous communities through their performance, choreography, teaching and facilitation.”

The independent dance sector is perceived as a communal, supportive and collaborative one in which adaptability is crucial to enable dancers to transition between projects and roles, and in which continual training and development is necessary to stay current in this varied field (Clarke, 1997; Rouhiainen, 2003). However, the unpredictable, non-linear nature of the career pathway means that it involves periods of instability and unemployment. Furthermore, dancers need to develop skills in administration, advocacy and marketing, and often take on additional employment to supplement their incomes which may restrict their performing and touring schedules (Clarke and Gibson, 1998; Kogan, 2002).

The hardships associated with a dance career have been well-documented, such as the arduous training without any guarantee of work on its completion, high risk of injury, and poor remuneration (e.g., Krasnow et al., 1994; Kogan, 2002). These hardships may be exacerbated by working in the independent dance sector, with its undefined roles, constantly changing working schedules, and lack of financial security. In the face of these varied challenges, it is valuable to understand why so many dancers pursue such an unstable career path. Although Clarke (1997) noted that they may not have a choice in this matter given the scarcity of full-time performing contracts, the fact that so many dancers do continue rather than simply pursue a more stable, alternate career means that the question remains important to address. This study sought to uncover what motivates independent dancers and how they navigate their careers.

Numerous theories of motivation suggest that humans are innately driven toward psychological growth and self-actualization. Self-actualization can be defined as the fulfilling of one’s potential, where life activities are engaging, meaningful, and congruent with one’s values (e.g., Maslow, 1954; Waterman, 1993). Authors generally agree that in order to self-actualize, a combination of particular aspects are required which tend to center around autonomy, satisfaction, and social relationships. Specifically, Seligman (2011) has recently argued that humans thrive when they experience positive emotion, engagement, positive relationships, meaning, and accomplishment. Ryff and Keyes (1995) work indicates that psychological growth results from autonomy, personal growth, self-acceptance, environmental mastery, and positive relationships. Similarly, self-determination theory (Deci and Ryan, 1985, 2000; Ryan and Deci, 2000) posits that individuals have an innate tendency toward growth and optimal psychological functioning which can be achieved once the basic needs of autonomy, competence and relatedness are satisfied. Satisfaction of these needs results in intrinsic motivation toward an activity, which occurs when one finds an activity to be inherently interesting and enjoyable in and of itself. Intrinsic motivation has been associated with greater enjoyment, satisfaction and likelihood that the individual will persist at the activity (e.g., Ryan and Deci, 2000).

Dance certainly appears to be an intrinsically motivating activity, which offers opportunities for psychological growth and self-actualization. Dancers have cited numerous intrinsic motives for engaging in dance such as self-expression, task mastery, emotional release and creativity (e.g., Alter, 1997; Stinson, 1997; Nieminen, 1998; Aujla et al., 2014). Dancers experience positive affect when the basic needs of autonomy, competence and relatedness are satisfied (Quested and Duda, 2010), and dance does appear to be well-placed to satisfy the basic needs. For example in terms of relatedness, young dancers are motivated to spend time with like-minded peers who act as both friends and inspirational co-creators (Aujla et al., 2014); in terms of autonomy, young dancers are more likely to persist at training when their teachers emphasize personal progression and effort (Aujla et al., 2015). Furthermore, dancers are passionate about their art (Aujla et al., 2014, 2015) and derive a large part of their identity from dance (Wainwright and Turner, 2004). Taken together, these findings suggest that the intrinsically motivating experiences dance can offer lead to enhanced commitment and potentially psychological growth, through creating and realizing one’s own ideas for example. Furthermore, these findings may help to explain why dancers continue in the face of hardships: young dancers report that they are aware of the challenges of a dance career but remain committed to their art because of the joy they derive from it (Aujla et al., 2014). However, while published research has provided valuable information on motivation among dancers, the participants were young dancers (Stinson, 1997; Aujla et al., 2014, 2015), pre-professional dancers in training (Nieminen, 1998; Quested and Duda, 2010) or professional ballet dancers, who typically work on long-term contracts (Wainwright and Turner, 2004). To date, no research has investigated the motives of independent contemporary dancers; this study aims to examine why they continue to persist in a sector that is notoriously insecure and unstable.

Independent dancers must cope with a portfolio career, managing multiple roles simultaneously often at different locations and with different groups of people. As such, they must not only maintain motivation in the face of uncertainty, but also organize various commitments, roles and demands, which may rely on key psychological characteristics. In sport, Alfermann and Stambulova (2007) define a career transition as a pivotal phase in an athletic career which comes with a specific set of demands that an athlete must cope with. Transitions can be micro, meso and macro in nature and may be normative (a predictable transition such as transferring from junior to senior level) or non-normative (an unpredictable transition such as dealing with injury or changing coach; Stambulova et al., 2009). Athletes may experience stress and uncertainty regarding the outcome of the transition, particularly when such transitions are non-normative, as these are hard to prepare for (Stambulova et al., 2009). Although the notion of career transition does not fully encompass the unique demands of the independent dance sector, in which a dancer may transition from one project to another relatively frequently, research suggests that successful transition depends upon whether the athlete’s resources (such as motivation and psychological characteristics) are sufficient to cope with the demands of, and barriers to, transition (Stambulova et al., 2009). Similarly, positive psychology researchers suggest that psychological well-being and optimal functioning is the balance point between an individual’s psychological, social and physical resources, and the challenges that they face (e.g., Dodge et al., 2012). It would be logical to assume that particular psychological characteristics and skills, such as determination and self-belief, may likewise aid independent dancers in their careers. Therefore, the second aim of this study was to investigate the extent to which psychological factors are employed by, and facilitate the career development of, independent dancers.

Psychological factors encompass both psychological characteristics and mental skills. While there is crossover in the use and definitions of these terms, for the purposes of this paper psychological characteristics are defined as features or qualities which may facilitate career development. These characteristics include commitment, optimism and self-belief (Gould and Maynard, 2009). Mental skills are defined by Vealey (2007) as those which enable peak performance and well-being through the regulation of thoughts, emotions and behaviors. Vealey (2007) identified four broad categories of skills: foundation skills, including self-awareness and self-confidence; performance skills which include perceptual-cognitive skills and attentional focus; personal development skills such as interpersonal competence and identity achievement; and team skills, including communication and leadership. In order to develop these skills, particular techniques can be employed, the most commonly reported being goal-setting, self-talk, imagery and arousal control. Regular and systematic practice of the techniques can enhance factors such as confidence, concentration and motivation, all of which can have a positive impact on performance (Vealey, 2007). The skills also determine how an individual interacts with their environment, such as taking advantage of opportunities (Abbott and Collins, 2004).

Evidence in sport suggests that both psychological characteristics and mental skills can differentiate elite athletes from their sub-elite counterparts (Morris, 2000). For example, elite athletes are characterized by their high levels of commitment and self-belief, mental preparation including the use of goal-setting and imagery, ability to focus and block out distractions, and effective anxiety management (e.g., Orlick and Partington, 1988; Gould et al., 2002; Mallett and Hanrahan, 2004; Gould and Maynard, 2009). Similarly, elite musicians are highly committed to their art, have a strong sense of self and set realistic goals (Talbot-Honeck and Orlick, 1998). Another similarity across the literature is that talented athletes and musicians appear to be generally optimistic, reporting high levels of positive thinking, and the ability to keep things in perspective (Kreiner-Phillips and Orlick, 1993; Talbot-Honeck and Orlick, 1998; Gould et al., 2002; MacNamara and Collins, 2011). Such an optimistic approach may be essential when entering the unpredictable independent dance sector whereby a dancer, however talented, has no guarantee of success. Although little dance research in the area exists, studies indicate that elite dancers use imagery more often and more effectively than non-elite dancers (Nordin and Cumming, 2005, 2006, 2007). There is also evidence that mental skills can be developed through training (e.g., Noh et al., 2007; Clark and Williamon, 2011), which is valuable given that some dancers assume that they cannot change factors such as their levels of self-confidence (Hanrahan, 1996) or performance anxiety (Walker and Nordin-Bates, 2010). Collectively these studies suggest that mental skills and techniques are associated with improved performance. However, few such studies considered the specific role mental skills may play in the longer-term career trajectory of a talented artist or athlete.

MacNamara et al. (2008, 2010a,b) and MacNamara and Collins (2011) broadened mental skills research to consider how particular factors, termed psychological characteristics of developing excellence (PCDEs), can help young athletes and musicians to negotiate talent development stages and the transition from learning environments to professional contexts. The PCDEs comprise many of the psychological characteristics and mental skills listed above, but also include the roles of vision, quality practice, and communication and social skills which are often necessary where career paths depend upon effective networking. This more extensive acknowledgement of attributes and skills appears valuable when considering career trajectories; however, PCDE research has been focused on talent development contexts. The current study sought to uncover whether broader factors such as those identified in PCDE research are employed by individuals already working in their chosen careers.

Overall, the aim of this study was to explore firstly dancers’ motivation to work independently, and secondly the psychological characteristics and skills that help dancers to succeed in the face of periods of unemployment, changing workloads and variety in a working week where they may adopt the role of performer, teacher, choreographer, and administrator in quick succession. Given the paucity of existing dance research, a qualitative approach was adopted to allow the most salient factors to come to the fore from the dancers’ own words. The rich descriptions yielded from qualitative designs enable researchers to explore a phenomenon from participants’ perspectives rather than from an *a priori* conceptualization (Patton, 2002; Krauss, 2005). Understanding more about the motivation and psychological characteristics and skills of independent dancers will help researchers and educators to both support working independent dancers, and to aid dancers in training to prepare for this challenging sector.

## Materials and Methods

### Participants

Participants were recruited via a web call-out in order to reach dancers who defined themselves as independent dancers. The web call-out outlined the aims, objectives and procedures of the study, and was hosted by an independent dance organization, ensuring targeted recruitment of an appropriate sample to provide rich data (Patton, 2002). Eleven female and three male independent contemporary dancers, with a mean age of 32.36 years (±6.05), volunteered to take part in the study. The dancers were professionals working in the industry and described their work as involving a combination of performance, choreography, teaching, and administration. Some took on additional work outside of the dance industry to supplement their income. The dancers were at various stages of their career, ranging from recent graduates to established artists. The participants had been involved in dance for 20.79 years (±7.58), although this varied greatly depending upon their age and stage of career.

Ten of the dancers had taken what could be considered a conventional route into the dance profession; that is, beginning to dance at a relatively young age (although among these, five described themselves as “late starters,” beginning dance between the ages of 14 and 18 years) then undertaking full-time dance training at a vocational or university institution. The training courses undertaken by the participants were varied in nature, including contemporary dance, commercial dance, performing arts, and choreography. The remaining four participants had taken less conventional routes into the profession. Two dancers pursued careers that were unrelated to dance before starting to dance at the age of 21 and 30 years respectively, while two participants had danced as children and young adults, but pursued other interests before returning to dance. Taken together, the dancers’ backgrounds suggest that there is no pre-determined route into the independent dance sector.

### Procedure

The study received ethical approval from a higher education ethics committee. Participants were given an information sheet prior to the study to familiarize themselves with the aims, objectives, and procedures of the research. Once participants were satisfied with this information, they gave informed consent in order to take part.

Interviews followed a semi-structured interview guide which was designed by the authors in consideration of previous research and gaps in the literature. The interview guide was split into four broad categories: introductory questions and background in dance; motivation to work independently and any changes in motivation over time (e.g., “Why did you persist in the independent sector when things were difficult?”); the advantages and disadvantages of working in the sector (e.g., “Can you describe the challenges you face in your career?”); and factors beyond their training that the dancers believed had helped them to succeed (e.g., “What sort of factors beyond training and opportunity would you say have helped you to get to this point?”; “How did you overcome challenges that you have faced?”). As this was the first study of its kind, the dancers were not asked explicitly whether psychological factors had helped them to navigate the sector, but rather which factors beyond their physical training had played a role, in order to allow any pertinent psychological factors to emerge inductively. Doing so would give an indication of whether or not dancers themselves identified psychological characteristics, factors or skills as being important in their careers. Before the interviews began, participants were informed that there were no right or wrong answers, and that they could take their time to consider their responses, ask questions or request clarifications if necessary. Probe questions were used to elicit further information where appropriate, and participants were given time at the end of the interview to add any additional information or ideas that related to the study topic. Participants continued to be recruited for the study and interviews conducted until theoretical saturation had been reached, i.e., to the point that no new information emerged (Patton, 2002).

Interviews were conducted by the second author at a time and place convenient to the participant. Three interviews were conducted via Skype due to difficulties in finding a mutually convenient time. The interviews lasted between 35 and 70 min, and the recordings were transcribed verbatim. Prior to the data collection phase, two pilot interviews were conducted with former independent dancers to assess the efficacy of the questions; as a result, some questions were modified or adapted.

### Analysis

The interview transcripts were uploaded to NVivo 10 qualitative analysis software. The transcripts were read and re-read to ensure familiarity, and then coded to generate specific meaning units. Given the lack of existing research in the area, the analysis was inductive in nature, allowing relevant meaning units to emerge from the data (Kvale, 1996). These meaning units were then organized and integrated to form larger categories (themes). Lower-order themes were arranged to create broader higher-order themes, and a hierarchy was created representing the relationship between these higher and lower order themes and the overarching research questions (Patton, 2002).

In order to enhance trustworthiness, the second author coded all of the transcripts, and the first author subsequently coded 15% of the transcripts independently. The independently generated codes were compared and debated to ensure agreement on labels, extent and amount of coding. The authors have a dance background, and understand dance concepts and terminology, enabling them not only to empathize with participants and use appropriate probes, but also to interpret the data from an informed perspective. Peer debriefing was conducted to discuss nascent themes with two objective experts who had experience of both the independent dance sector and the research process (Creswell and Miller, 2000). The discussion aided with interpretation of the lower order themes, and provided further contextual understanding once the next stage of analysis began. The authors independently organized the lower order themes into hierarchies, and the two versions of the hierarchy were contrasted and debated with reference to the codes themselves and existing literature until a final version was agreed upon (Patton, 2002).

Following this, member checking was conducted by offering the participants the opportunity to review and discuss the final versions of the hierarchy (Creswell and Miller, 2000). This took the format of an informal round table discussion, enabling the participants to confirm that the conclusions were just and accurate. Finally, quotations are included in the Results to illustrate specific lower order themes, and to allow readers to form their own interpretations (Sparkes, 1998). Quotations are labeled according to sex and the order in which the interviews were conducted (e.g., M1).

## Results

**FIGURE 1:**
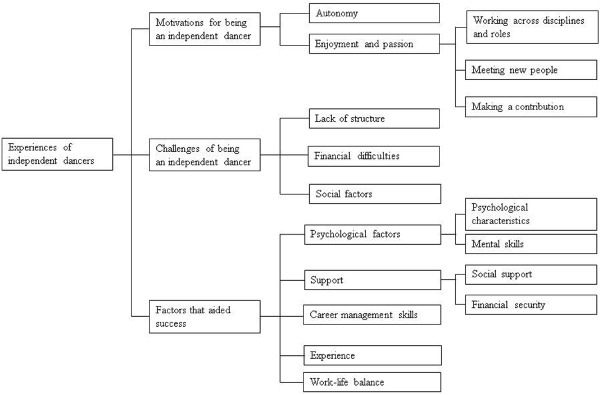
**Hierarchical representation of the results**.

Three higher order themes emerged that created the overall hierarchy of motivation and psychological factors among independent dancers: motivations for being an independent dancer; challenges of being an independent dancer; and factors that aided success in the sector. These themes, and the lower order themes which contributed to them, are outlined in turn below, and are also represented in Figure 1.

### Motivations for Being an Independent Dancer

This theme was created from data regarding the dancers’ reasons for working independently. These fell into two key categories: autonomy; and enjoyment and passion.

#### Autonomy

One of the main reasons for working independently that emerged from the interviews was the opportunity to be autonomous. The dancers appreciated the freedom the role afforded to develop as dancers and artists: “I’ve been really lucky trying to exist in a place where I’m hopefully employed for being me, and being the best me that I can be” (M9). Dancers were able to forge their own identity and work toward their own vision. This was perceived as being different to working for a company, in which dancers might lose a sense of their own identity:

“I had a perfectly fine job at the theatre and everything is taken care of, but I feel artistically there are limits… It’s very much restrictive in terms of what you can do…as a freelance dancer, or artist in general you can basically have your own vision and I think that’s the biggest difference in terms of fulfillment” (M1).

“I was 18 and I did a combined arts degree and it didn’t work for me, it didn’t suit me and I remember thinking then, I don’t know why I’m doing this because I don’t want to do auditions anyway. I want to make my own work. I had a lot of ideas and just felt that if I worked with a company I wouldn’t be able to do that” (F13).

Some dancers explained that working for a company involved too many boundaries, whereas working independently meant that dancers were free to make their own choices: artistic ones (such as making creative or choreographic decisions) and career choices (such as being able to choose which project/s to pursue, and roles within a particular project), allowing dancers to work on their own terms:

“…being able to choose what I do and when I do it to a certain extent…So when I enter a new agreement with someone I can define from the start exactly what it is I want from them, what they want from me and it can be a complete negotiation, whereas when you’re signing on a contract you are fitting a brief, and some of it might be good, some of it might be bad but you don’t have as much choice” (F3).

Similarly, working flexibly was perceived to be important, as it engendered autonomy of working patterns and routines, which is exemplified in the following quote: “The managing of my own time means…I can work from home, I can work wherever I wish to work sometimes” (F2). For some dancers, part of this freedom and autonomy also meant that they could indulge their love of travel by touring their work, and they also enjoyed the spontaneity that came with the job: “I think the spontaneity of being able to make decisions on your on you own behalf is fantastic. I don’t have to go through a board of directors or ask anybody’s permission” (F13). Taken together, such freedom, choice and independence in relation to career development and artistic decisions were critical factors in the dancers’ reasons for working independently.

#### Enjoyment and Passion

The dancers were incredibly passionate about dance in general and their work as independent dancers. They suggested that they had to dance; that some inner force drove them, for example: “There is just absolutely nothing that I enjoy as much as this… it makes me whole [laughs] … I’m so glad I made the choice [to work independently], it’s the best choice I’ve ever made for my career” (F14).

Working in the independent sector in particular was perceived as extremely fulfilling. The participants found enjoyment, creativity, and variety in their work: “I think I’ve always taken great pleasure in diversity and enjoying a diverse range of things, working in lots of different areas” (M9). Another dancer explained: “I love meeting new people and I love being challenged every day and I like having something different from week to week” (F3).

As such the dancers valued not only the autonomy of their careers, but also the constantly evolving nature of their work and the varied lifestyle this entailed. Three key reasons for enjoying working independently emerged: working across disciplines and roles; meeting new people; and making a contribution.

***Working across disciplines and roles***

Dancers appreciated being able to fulfill several roles, such as performer, choreographer and teacher, and working across a range of artistic disciplines, within one career. There was an understanding that these roles informed each another and led to creative growth: “I love how dance connects with other theories and other, other art forms …you’re constantly engaging with new things” (F3).

***Meeting new people***

The participants described the enjoyable advantages of working with a range of different people in terms of new insights and ideas, collaboration, inspiration, making new contacts and staying current in the field. For example, one dancer commented on, “the diversity of people that I have worked with and continue to work with, in terms of stimulation and creative growth” (F2). This network of contacts was not only an enjoyable element of their work, but also contributed to the dancers’ success, as is explored in a subsequent section.

***Making a contribution***

Several participants discussed the desire to, “make a contribution, and for that contribution to be recognized” (M11). The autonomy and creativity that came with the independent role offered dancers the opportunity to make a valued contribution to their field. For some dancers, the chance to contribute meant choreographic innovation; for others it entailed making audiences or participants happy, and changing people’s lives. As an illustration, one dancer explained: “it’s this joy thing, trying to get people to connect with that whether or not the watching dance or participating or the joy of creating a dance, so I think it’s trying to be – I kind of joke a bit that I’m a dance missionary trying to spread the word of dance” (M9). Therefore, a number of participants were motivated by a desire to help others experience the joy and fulfillment they themselves gained from dancing.

### Challenges of Being an Independent Dancer

While the participants reported several important reasons for working independently, they also noted a number of challenges which made the role difficult. These included: lack of structure; financial difficulties; and social factors. Importantly however, the dancers concurred that the advantages outweighed the disadvantages: “I think the advantages over weigh it because you get to do what you love and there’s no kind of – well there are boundaries but you can push it as far as you want really” (F6). This suggests that the autonomy and enjoyment the dancers derived from their work was sufficient to overcome its associated disadvantages.

#### Lack of Structure

Dancers described the uncertainty of their work and the difficulties around planning their careers and lives in general. Some periods of their lives were very busy, while others were much quieter. These shifting work patterns had divergent emotional effects: when dancers were juggling a range of roles and projects, some felt guilty that they were, “feeling that I spread myself so thinly over many different things and I can’t really commit to one thing. And the feeling of always being behind with work” (F4). On the other hand, quieter periods could be worrisome: “you are in and out of work, and yeah it’s not very stable. Like I’ve just come out of another contract and then you have a bit of a panic, like what am I going to do now” (F6). For some dancers this lack of security could cause problems with anxiety, self-confidence and motivation:

“…the gaps and the not knowing and the anxiety of not knowing where you are going. Or perhaps because you don’t have that full structure maybe you lose motivation at times, or confidence… it’s a constant wave, a massive rollercoaster. And right now I’m in a bit of a dip, and it’s really a quite unpleasant feeling” (F12).

Several dancers had created some structure and stability in their working lives by taking on contracted part-time teaching work, around which they continued to freelance. However, this was not without problems as the regularity of teaching, designed around academic terms, could restrict project-based work. Furthermore, the nature of independent work often relies on informal contracts and agreements between dancers. This lack of formality made it difficult to negotiate work: “…people promising me work but then not getting the contract and the confirmation through and they weren’t friends so it was just their word. And it was 2 weeks, 3 weeks before and obviously if that work falls through…So that emotional worry” (F4).

***Work-life balance***

It also became apparent during the interviews that part of the problem with a lack of regular working hours was finding a satisfying work-life balance. The participants described difficulties and guilt with being away from family and friends, and missing out on important events such as weddings, due to unsociable hours and touring. Maintaining a social life on a low wage was often difficult, and non-dancing friends and family did not always understand the nature of the work and why it made the dancers less available.

“…it’s hard to maintain a social balance in your life…A lot of my friends aren’t dancers…they have a 9 to 5 job and they know when they are available every week to meet and so there are times when you get invited to an event and my stock answer used to be like – actually I’m in Wales…Then I think people forget to ask you then when you’re back, so then it becomes a bit of a – oh we didn’t know you were about so we stopped inviting you to things” (F10).

Some participants recognized how tiring the work could be: “You get to travel quite a lot which is really nice…but sometimes it’s quite exhausting, sometimes I wish I travelled less” (F5). The lack of structure also made it difficult for dancers to plan activities outside of work such as holidays, although this was often because dancers were anxious about potentially missing out on interesting opportunities by taking time off: “It would be really nice to be able to plan a year, and plan in a holiday but it would come at a cost of therefore not necessarily being available to do interesting stuff when it turns up” (F2). As that quote suggests, some dancers struggled to manage their time effectively, and most had not yet achieved their ideal work-life balance. One dancer in particular described an all-or-nothing approach to her work:

“I made a decision not to have children because it was children or the work. I couldn’t see how you could do both and I didn’t want to be in a situation I guess where I may have had children and resented not being able to do – you know, I am an artist, that’s what I am and I can’t really not do it… I mean I have very little time at home. I’m at work all the time so I don’t, this sounds really sad, but I don’t really have a social life” (F13).

#### Financial Difficulties

Financial factors were discussed as a challenge of working independently, particularly as they pertained to stability. When starting out, dancers often had to volunteer or take unpaid jobs in order to gain experience to help them find further work. However, this expectation that dancers would work for free was not a problem exclusive to younger or inexperienced dancers, as one participant noted: “I’m 40 next year, and I still get people ringing me asking me to do things for free… I’ve trained for 5 years; I’ve been working for 16, like, would you ask a vet to do it for free?!” (F7). Even once dancers had gained paid employment, this was often poorly remunerated, and there were few full-time contracts in the sector. If dancers were not seeking paid contracts, they often applied for funding from arts, charities or government bodies to fund their projects but this also tended to be relatively short-term. As one dancer explained: “…financial cash flow is a major disadvantage, having no financial security in that way but I kind of feel like I’ve done it for so long now it’s just how it is and just try not to let it stress me out” (F8). The lack of a regular income not only made it difficult to pay for crucial expenses such as accommodation costs and bills, it also made it harder for dancers to attend regular class (which was important for their technical and artistic development) and auditions and networking opportunities (which were important for getting work), and as noted above interfered with their social lives. One male dancer also highlighted the financial implications of starting a family, expressing, “can I support them, can I really deliver, or, like, provide for them?” (M1).

Interestingly, it emerged that some dancers and choreographers were reluctant to talk about money, firstly because it appeared to compromise their artistic integrity: “no one likes talking about money, whether it’s institutions or a funded company. You feel like you’ve got to maintain artistic integrity…I think there’s this notion that it’s about producing your art, so it’s about your artistic identity and the fulfilment of the project. But then it’s hard to earn anything [laughs]” (F10). Secondly, this reluctance was caused by guilt or a lack of confidence in having to apply for funding for consecutive projects: “as an independent artist if you ask for money you always have the perception of being, ‘OK, I’m sorry I need money’. That shouldn’t be like that. It’s actually ‘OK, I have something amazing to do, please give me some money”’ (M1). These issues further confounded difficulties with securing adequate pay. The dancers also noted that due to the nature of their work, there was no paid holiday, pension scheme or health insurance.

#### Social Factors

Finally, while the dancers discussed difficulties with maintaining a social life alongside their careers, they also described social challenges that were specific to their work. Firstly, the autonomy and freedom of the career also meant there was no real sense of being part of a team: “you are working on your own a lot and that can become very isolating” (F7). This coupled with variable working hours meant that although building a social network in the industry was important, it was sometimes difficult to maintain: “…you’ve built up this whole bank of people there, and try to keep in contact with them all, and it’s been so difficult” (F3). Alongside feelings of isolation, the lack of formality in some working relationships was not only difficult in terms of anxiety around pay and contracts, but also around loyalty and allegiances: “the loyalty, I suppose, of some working relationships has also burned me in the past where I’ve been offered something and then it’s been taken back because there’s been no contract” (F2).

### Factors that Aided Success

Although the focus of this study was on psychological factors, the participants discussed a broad range of factors which they believed had helped them to negotiate and succeed in the independent dance sector, and overcome the challenges listed above. These findings created the following themes: psychological factors; support; career management skills; experience; and work-life balance.

#### Psychological Factors

The dancers reported a range of psychological characteristics and mental skills that aided their success.

***Psychological characteristics***

Firstly, dancers noted the importance of being proactive, open, enthusiastic, and able to make the most of opportunities and try new things. This positive, proactive stance represented the ability to create opportunities, and a willingness to take on new challenges, because they offered dancers novel experiences and artistic growth. For example: “it’s still scary but I think there’s part of me that’s going but I want to work with someone else now, get someone else to feed and change my – because when a different choreographer comes in…you learn their new material and go – ah this is a new challenge and I think, yeah, that’s exciting” (F10). As such the dancers were generally optimistic; one particular dancer had an explicitly optimistic outlook and was keen to put his work into perspective: while he loved his job and derived a great deal of joy from it, he also expressed:

“as much as I worry about things and I have doubts and insecurities, I think we all do, and I try to be as positive as possible and I think that helps to go, ‘everything will be alright and if it’s not alright it’s not the end’… it’s only dancing. And I think we kind of forget that. You can take it really seriously and you just go – ok I’m lucky to be doing this” (M9).

Interestingly, some dancers were perhaps too modest in claiming that their careers had “just happened,” but taken together the data revealed that these participants were determined to make the most of opportunities available to them. This was supported by a strong commitment and dedication to their work, discipline and a good work ethic: “I work really hard to make things happen” (F8). Alongside these factors were tenacity, patience and perseverance: “I know a lot of people that have taken quite a long time to get in to it…if you don’t go to auditions you’re not going to get the jobs…When you kind of stick around, if you are present there’s more of a chance the right thing will find you” (M9). The dancers perceived success to be reliant upon the willingness to work hard and stay committed even in the face of setbacks.

***Mental skills***

The dancers discussed three specific mental skills which appeared to aid their success. Confidence was reported as an important skill that helped dancers to maintain their open and committed approach. As one dancer explained: “…often things aren’t as scary as they seem, and trying to have the confidence that you have the skills to do it” (M9). Many dancers reflected that they had become more confident with their varied roles over time and with experience. This helped them to remain confident and optimistic about their careers: “continuing to believe that you are capable of getting work, and enjoying it, and developing as a dancer because of it” (F14).

Self-awareness was discussed, in maintaining artistic integrity and a commitment to one’s own vision, and understanding one’s own strengths and weaknesses: “you can really get to discover your own capabilities and then challenge yourself accordingly” (F14). Self-reflection was another skill deemed to help dancers to succeed: “I’ll take all the work I’ve done in the studio in a week and I think how I’d write that to let an outside person know what it’s like to spend a day doing this and the route that we’ve gone on…you’re kind of getting a lot reflectively from that…it does feed into the studio practice as well” (F10).

#### Support

Two forms of support emerged as being critical in helping the dancers to succeed in the independent sector: social support and financial security.

***Social support***

This category was split into two sub-categories, the first of which was support from the dance community. This included support from dance tutors and lecturers during training and beyond, peer support and mentoring, formal mentorships where they were available, and collaborating with other dancers to “play” regardless of whether that became a project. “I’ll quite often get together with a network of people who are in a similar position, and we might just come together in the studio and play for a couple of hours. Or we might just chat, or we might just go for a coffee” (F7). This sense of community appeared crucial in combatting the isolating elements of the career, and had to be consciously created and maintained:

“I guess you create for yourself a little club of people, if you don’t have a support team as in a workplace, with people for yourself. There’s four or five people I meet every four or five months and I find it really encouraging and really helps me. I just ask for their advice for things…Yeah you create your own little support network” (F4).

In order to capitalize on such support, the dancers recognized that good people skills enabled effective networking, communication during projects, and that generally being friendly and treating others well smoothed the working process and made it more likely that they would be recommended for other projects: “I think I am a good people person, that is one of my – I’m very sociable and I like people… I think being sociable and open is a really important thing” (F12).

The second kind of support came from friends, family and partners who were not involved in dance. This involved the kind of financial support noted in the next section, but also emotional support, motivation, and assistance with specific skills such as marketing. For example, one dancer explained that her parents and husband encouraged her and modelled a strong work ethic: “There’s a sense of nurturing in my home environment that’s made me succeed or work hard” (F8).

***Financial security***

All of the dancers expressed that financial stability was paramount to their working lives and development. Some dancers had generally achieved financial stability through paid contracts and successful funding bids, although they still cited other forms of support such as those listed below. Other dancers tended to achieve stability through one of two means: financial support from partners or parents; and taking on non-dance related jobs to supplement their income. The former could mean accepting financial support from a partner with a more traditional, permanent job: “I am very fortunate that my husband has a proper job [laughs], he has a good job, so at times when I’m not earning a lot, that’s still kind of ok with us” (F14). For younger dancers this often meant living at home with parents to reduce accommodation costs. For example: “…because I still live at home with my parents, so I couldn’t keep going unless I was there and I had their support, because of that sense of sometimes I earn a lot of money at once and sometimes having to wait for people to pay up…that’s a big thing because it just gives me more chance to achieve things, rather than constantly be thinking I’ve got to pay rent” (F10).

Dancers also described finding employment that was not related to dance at times, which enabled dancers to continue pursuing an independent career: “There’s still this real stigma attached with ‘oh gosh I’m just working in a bar’, or ‘I’m just working in Tesco’. But if that enables them to continue to be freelance for the time it takes for them to get the job that they want, I would encourage that and say ‘stick at it”’ (F14).

#### Career Management Skills

Given that independent dancers must manage and represent themselves, at least some entrepreneurial skills, as well as business competencies such as presentation, communication and writing skills, are necessary. Some participants felt that these aspects of the role were neglected during their training, and should be provided as part of dance training rather than dancers having to develop these skills while they work. For instance, one dancer described the difficulty in translating artistic concepts to business audiences when applying for funding: “I didn’t have the skills to present my practice well” (M11). However, a number of the participants actively sought opportunities to develop these skills, for example: “…next week I’m going to a woman’s networking meeting, in terms of the business side of things…I’ve been attending quite a lot of local creative learning network meetings, and things organized by the Arts and Heritage Alliance” (F10).

Additional career management skills such as time management, organization and the ability to multi-task were cited, although not all dancers felt that they had developed these to a good degree: “the main thing I’ve noticed that shifting into self-managing and I suppose there’s that discipline and rigor. I know that’s something I’m not very good at” (M9). The self-management and discipline required to work independently appeared to develop with time and experience; dancers newer to the sector found this particularly hard when starting out and felt that having someone else set boundaries could be helpful. Finally, the importance of technology, in particular portable laptops, was noted for dancers who do not have an office base or a typical working schedule, while one participant highlighted how having a driving license and car was essential for getting to and from different jobs and venues.

#### Experience

The older or more established dancers agreed that the more experienced and renowned they became, the more likely they were to be busy, in demand, and financially stable. The dancers had accrued valuable work experience in several different roles such as working on varied projects with different choreographers and dancers, teaching, and administration, which enhanced their employability. As such, these dancers felt that working independently had become easier over time, and that they were more aware that certain patterns of work tended to recur over the years: “It changes a lot but actually there are, kind of, patterns and cycles of repetition…if I look back” (F2). Recognizing at least some recurring work patterns may have served to reassure dancers in times of uncertainty.

Some dancers felt that they had had good preparation for the sector as part of their training, such as performance platforms that modeled professional practice: “the lecturers there were creating opportunities, like platforms for us to showcase our work, and things like that. And they would help with, if I had an audition or I wasn’t sure of something I could always go to them” (F6). However, this was not universally the case and a number of participants felt that they had not been sufficiently prepared for independent work by their training experience. Specifically, some dancers noted that there had been little training in career management skills, skills for additional roles such as teaching, or a general sense of what to expect when transitioning into the profession. “…my training was all about learning technique and we did a bit on writing CVs and bits and bobs but not massively on how to get yourself out there and progress your career particularly” (F10). Therefore for these dancers, experience and learning “on the job” was the only way to develop the additional skills required in the sector.

#### Work-Life Balance

Achieving a satisfactory work-life balance could be challenging, but the dancers recognized that this was important not only for their general health and well-being, but also artistically to shift their perspective and find novel solutions to problems:

“being able to see things from a different angle or just to go a different way or not take things at face value, think around the problem and approach it from different side, and having the space and the time to step back and do that. And to not always be rushing round. I think that’s the time I get most of my ideas, the kind of new ideas that excite me” (F4).

Most of the participants aimed to spend more time with their partner or friends. Dancers described prioritizing social events and activities over work at times by weighing up what could be gained from a work opportunity and acting accordingly. Although some dancers struggled to make time for a holiday, others had come to realize that taking time off was essential: “This summer I realized, I think in June, that I really needed to stop. I was really tired, I’d been running from one project to another and fundamentally that means that you don’t have time to reflect or time to properly plan” (M9).

A number of dancers explained that meeting their partner, who was not involved in dance, had helped them to achieve a better work-life balance. For example, one dancer’s partner had changed her perspective: “when I met my husband, I kind of got a reality check, that actually work is not your life, human beings and all of that is much more important, and friends and stuff” (F8). Another participant had turned down a job abroad to be with her partner, while two dancers stated that they never worked at weekends otherwise “my husband would divorce me [laughs]!” (F8).

## Discussion

The aim of this study was to better understand dancers’ motives to work independently, and to uncover the psychological characteristics and mental skills that enabled dancers to successfully negotiate this challenging sector. Given the unpredictable and non-linear nature of the dancers’ careers, it could be suggested that they dealt with non-normative micro transitions on a regular basis (Stambulova et al., 2009) which at times caused stress and anxiety (Alfermann and Stambulova, 2007). The dancers’ ability to negotiate these transitions successfully appeared to rely on their motivation and psychological characteristics and skills (Abbott and Collins, 2004; Stambulova et al., 2009), and, due to the exploratory and inductive nature of the research, additional factors such as social support, experience, and work-life balance. These findings provide support for a number of previous studies in areas including motivation (e.g., Aujla et al., 2014, 2015), mental skills (e.g., Vealey, 2007) and PCDEs (MacNamara et al., 2010a,b, 2008; MacNamara and Collins, 2011), and represent the first time that such factors have been investigated simultaneously in dance. The key findings will now be discussed in turn in relation to relevant literature.

The dancers reported a number of reasons for working independently, which included a strong love and passion for dance. Previous research has indicated that dancers are passionate about dancing, and that this passion is an important component of their commitment to dance (Aujla et al., 2014, 2015). Just like young dancers and those in training (Alter, 1997; Nieminen, 1998; Aujla et al., 2014), the participants enjoyed the intrinsically motivating characteristics of dance such as creativity and working across artistic disciplines. However, the current study has moved beyond previous studies of motivation in dance (e.g., Alter, 1997; Aujla et al., 2014) to identify motives to work independently. Similar to reports from professional dancers (Critien and Ollis, 2006) and talented athletes and musicians (e.g., MacNamara et al., 2010b), the participants were highly committed to dance, and to continual improvement and development. The opportunity to take on varied roles enabled the dancers to learn new skills and remain motivated.

One of the key reasons why the dancers enjoyed working independently was the autonomy it afforded them. Working independently meant that the dancers were free to pursue their own artistic interests and visions, could engage with the sector spontaneously, and enjoyed freedom and flexibility around their working schedules. According to self-determination theory, autonomy is one of the fundamental basic needs which underpins intrinsic motivation (Deci and Ryan, 1985, 2000; Ryan and Deci, 2000). Autonomy was particularly important to these dancers, and it appears that working in the independent sector offers one of the best ways in which to work autonomously in the dance domain.

Another element of independent working that the dancers enjoyed was the opportunity to work in different roles, disciplines, and with different people. The dancers concurred with Clarke (1997) that the independent dance sector was a creative and supportive community. Similarly, young dancers have discussed the importance of their dancing peers for emotional support, creative collaboration and inspiration (Aujla et al., 2014), and these findings further highlight the importance of relatedness as a factor that facilitates intrinsic motivation (e.g., Ryan and Deci, 2000). Moreover, working in different roles and with different people offered the dancers numerous opportunities to develop as performers, teachers and choreographers.

The dancers felt fulfilled by their work but also that it gave them room to grow as artists. Many of them also valued the chance to make a contribution to the wider society. Interpreted through the lens of positive psychology, it appears that working independently offered dancers numerous opportunities to fulfill their potential, enhance their psychological well-being, and self-actualize. For instance, autonomy and environmental mastery are important elements of psychological well-being (Ryff and Keyes, 1995), and could be represented by the autonomy and sense of being in control of one’s identity and future that the dancers described. The dancers enjoyed pursuing their artistic visions and aimed to make a contribution to society, similar to notions of engagement and meaning highlighted by Seligman (2011) in his work on human thriving, and Shah and Marks’ (2004) definitions of optimal well-being. Positive and meaningful social relationships are also central to a number of motivation and self-actualization theories (Ryff and Keyes, 1995; Ryan and Deci, 2000; Seligman, 2011). Taken together, it could be argued that working in the independent sector provides dancers with several opportunities to experience psychological growth, well-being and fulfillment from an activity that they find important, meaningful, and joyful.

Despite the many opportunities the independent dance sector afforded for psychological growth, a number of these benefits could also be perceived as disadvantages. The autonomy and freedom that came with working independently resulted in a lack of structure, which made it difficult for dancers to plan their careers, lives and finances. Some dancers highlighted the emotional impact of this, in terms of lessening their motivation and self-confidence, and increasing their anxiety. The uncertainty and insecurity of the role left these dancers feeling isolated and unsure of themselves during quiet periods, perhaps because a lack of work prevented dancers from feeling competent and related (e.g., Deci and Ryan, 2000). Moreover, the autonomy and variety in schedules led to difficulties with work-life balance. Firstly, dancers struggled to switch off, which may occur when individuals are involved in a vocation that takes up a large proportion of their identity (Wainwright and Turner, 2004). Secondly, the constantly changing schedules and unsociable hours made it difficult for dancers to establish a structure or routine. The stress and anxiety caused by financial concerns could have also contributed to unsatisfactory work-life balances as some dancers may have had to keep working to make a living.

In order to overcome the difficulties associated with the independent career, the participants described a number of characteristics, skills, and support networks which aided their success. These findings provide support for recent conceptualizations within the positive psychology literature in that optimal functioning and well-being appear to rely on the balance between the challenges an individual faces, and their psychological, physical and social resources (Dodge et al., 2012). Firstly, the dancers explained that being proactive, open to new challenges, and willing to create or make the most of opportunities was essential to working independently. This may have been related to the dancers’ optimism, as many of them believed that “things would work out.” Some dancers also tried to keep their work in perspective to prevent the unpredictable nature of the work from causing anxiety. Previous research has similarly found that elite athletes and musicians are characterized by an optimistic and positive outlook, and the ability to retain some sense of perspective (Kreiner-Phillips and Orlick, 1993; Talbot-Honeck and Orlick, 1998; Gould et al., 2002; MacNamara and Collins, 2011). In the face of numerous challenges, such an outlook may be essential to help talented individuals continue to strive toward success rather than quit (Scheier and Carver, 1985). Experience in the independent sector, and the ability to identify certain working patterns over time, could also have helped the dancers to remain optimistic.

Such optimism and openness may have been underpinned by the dancers’ commitment to their work. Many of them explained that they simply could not imagine doing anything else. As noted earlier, the dancers were intrinsically motivated and passionate about dance; the sheer joy, meaning and fulfillment they derived from their work meant that, like elite athletes and musicians, they remained highly committed to their art, persevered in the face of hardships, and applied a strong work ethic to help them continue to develop (e.g., Orlick and Partington, 1988; Talbot-Honeck and Orlick, 1998). Some dancers also explained that confidence helped them to persist. While the sport literature indicates that self-confidence is associated with elite performance (e.g., Gould et al., 2002; Vealey, 2007), perhaps a more appropriate term in the context of this study is self-belief (Gould and Maynard, 2009), which represents a broader trust in one’s abilities, and an expectation of continued opportunities or success.

Two final related mental skills mentioned by some of the participants were self-awareness and self-reflection. Vealey (2007) identified self-awareness as a foundation mental skill, and several authors have found that elite athletes evaluate past performances to elicit future improvements (e.g., Orlick and Partington, 1988; MacNamara et al., 2010b). Understanding their strengths and weaknesses may have helped the dancers to prioritize particular projects that played to their strengths, or select ones which challenged them and helped them to improve. Reflecting on a day’s work in the studio, and broader reflections during quiet periods or time off, appeared to give dancers the time and space they needed to contemplate, problem-solve, and find new perspectives. All of these factors could clearly have a positive impact on their artistic practice both within and between projects. Taken together, the findings of the current study indicate that psychological characteristics and mental skills identified in previous literature are not only important in relation to performance enhancement (e.g., Gould et al., 2002) and talent development (e.g., Abbott and Collins, 2004), but also play a significant role in relation to longer-term career development and trajectories.

The dancers discussed the importance of experience, in terms of enhancing their employability and self-awareness, and cited a number of career management skills that also helped them to succeed. These skills, including administration, accounting, organization and time management, are essential for independent dancers who have to manage and represent themselves (Clarke and Gibson, 1998; Kogan, 2002). Such skills aided in searching for work, applying for funding, and juggling many demands. Given the importance of social networks in the independent sector (Clarke, 1997; Rouhiainen, 2003) it is unsurprising that the dancers also noted the importance of social skills for career progression in terms of making contacts and creating positive working relationships. Vealey (2007) discussed interpersonal competence and communication as important mental skills, while in their PCDE research, MacNamara et al. (2008) found social skills to be important among young musicians, whose transition from talent development contexts to the profession often relied upon effective networking. Additionally, social skills enabled dancers to call on social support when needed, either from other dancers to collaborate, create and “play,” or from non-dancers for assistance with skills such as marketing. These networks were just as crucial during quiet periods to reduce feelings of isolation.

Social support from non-dancers appeared equally important. The dancers’ family and partners provided emotional support and encouragement, and may have helped the participants to improve their work-life balance and retain a sense of perspective. Moreover, financial stability for some dancers could only be achieved with the support of partners and family. However, their work in the independent dance sector was so important to the participants’ identities and sense of fulfillment that they continued to pursue their artistic visions and aspirations. Talent development research has highlighted the importance of social support from non-dancing family and friends in relatively stable environments (e.g., Aujla et al., 2014); the results of the current study indicate that social support from family and partners continues to be important while working in the unpredictable independent dance sector.

### Implications

The participants reported using a range of mental and career management skills, which they had developed intuitively and gradually in response to the demands of the sector. However, some dancers felt that they had not developed them to a sufficient degree. In the arts, studies have similarly reported that dancers have created their own strategies, for example in developing effective imagery (e.g., Nordin and Cumming, 2006), and coping with performance anxiety (e.g., Walker and Nordin-Bates, 2010). However, there is evidence that artists can be taught mental skills systematically with positive results. As an illustration, musicians reported increased confidence, self-awareness, and healthy perspectives toward music-making following a 9-week mental skills intervention (Clark and Williamon, 2011). Embedding these skills in dance training may help dancers to transition into the sector with greater ease.

The only attributes dancers did not need training in were motivation and commitment. In another qualitative study, Hanrahan (1996) similarly found that motivation was not a problem among dancers; however, such high levels of commitment and dedication may interfere with dancers’ ability to achieve a satisfactory work-life balance and it appears that career management skills, such as time management, may be beneficial in this regard. Dancers in training, and those in the profession, could also benefit from specific workshops or seminars around funding applications, marketing, and communication skills, to ensure they have the necessary competencies to support, manage and promote themselves. It could also be beneficial for dance organizations to provide forums and space to facilitate networks among dancers to reduce their sense of isolation, strengthen existing relationships, and forge new ones. However, it should be noted that the results of this study cannot be generalized to the whole dance population as the independent sector has unique demands which are not typically found in other sectors of the industry such as ballet companies.

## Conclusion

Overall, independent dancers are passionate, highly motivated and committed individuals whose love of dance and the opportunities it offers for growth and fulfillment are sufficient to overcome the numerous hardships associated with an independent career. The extent to which such hardships are perceived or experienced as disadvantages, and are overcome successfully, may depend upon dancers’ psychological characteristics, in particular the adoption of an optimistic outlook, their use of relevant mental skills, and the amount of social support and financial stability available to them. Furthermore, while factors such as psychological characteristics, mental skills, social support, and career management skills are critical elements of talent development, the current study indicates that they also play a crucial role in ongoing career development once dancers are working in their chosen sector.

### Conflict of Interest Statement

The authors declare that the research was conducted in the absence of any commercial or financial relationships that could be construed as a potential conflict of interest.
